# Discovering Putative Prion-Like Proteins in *Plasmodium falciparum*: A Computational and Experimental Analysis

**DOI:** 10.3389/fmicb.2018.01737

**Published:** 2018-08-07

**Authors:** Irantzu Pallarès, Natalia S. de Groot, Valentín Iglesias, Ricardo Sant’Anna, Arnau Biosca, Xavier Fernàndez-Busquets, Salvador Ventura

**Affiliations:** ^1^Institut de Biotecnologia i de Biomedicina, Universitat Autònoma de Barcelona, Barcelona, Spain; ^2^Departament de Bioquímica i Biologia Molecular, Universitat Autònoma de Barcelona, Barcelona, Spain; ^3^Centre for Genomic Regulation, The Barcelona Institute of Science and Technology, Barcelona, Spain; ^4^Universitat Pompeu Fabra, Barcelona, Spain; ^5^Nanomalaria Group, Institute for Bioengineering of Catalonia, The Barcelona Institute of Science and Technology, Barcelona, Spain; ^6^Barcelona Institute for Global Health, Barcelona Centre for International Health Research (Hospital Clínic – Universitat de Barcelona), Barcelona, Spain; ^7^Institute of Nanoscience and Nanotechnology, University of Barcelona, Barcelona, Spain

**Keywords:** *Plasmodium*, protein aggregation, amyloid, prion, Q/N-rich sequences, protein disorder

## Abstract

Prions are a singular subset of proteins able to switch between a soluble conformation and a self-perpetuating amyloid state. Traditionally associated with neurodegenerative diseases, increasing evidence indicates that organisms exploit prion-like mechanisms for beneficial purposes. The ability to transit between conformations is encoded in the so-called prion domains, long disordered regions usually enriched in glutamine/asparagine residues. Interestingly, *Plasmodium falciparum*, the parasite that causes the most virulent form of malaria, is exceptionally rich in proteins bearing long Q/N-rich sequence stretches, accounting for roughly 30% of the proteome. This biased composition suggests that these protein regions might correspond to prion-like domains (PrLDs) and potentially form amyloid assemblies. To investigate this possibility, we performed a stringent computational survey for Q/N-rich PrLDs on *P. falciparum*. Our data indicate that ∼10% of *P. falciparum* protein sequences have prionic signatures, and that this subproteome is enriched in regulatory proteins, such as transcription factors and RNA-binding proteins. Furthermore, we experimentally demonstrate for several of the identified PrLDs that, despite their disordered nature, they contain inner short sequences able to spontaneously self-assemble into amyloid-like structures. Although the ability of these sequences to nucleate the conformational conversion of the respective full-length proteins should still be demonstrated, our analysis suggests that, as previously described for other organisms, prion-like proteins might also play a functional role in *P. falciparum*.

## Introduction

Malaria caused approximately 445,000 deaths in 2016 and in the latest World Malaria Report (November 2017) the number of cases was estimated to be as many as 216 million. Although the global response to malaria is considered one of the world’s great public health achievements, the spread of resistance against anti-malarial drugs and insecticides, has stalled the incidence and mortality decline since 2014.

*Plasmodium falciparum* is the species responsible for 85% of the malaria cases, causing the most severe form of the disease. The complete sequencing of *P. falciparum* genome has revealed some specific features that may shed light onto the biology and biochemistry of this deadly parasite (Gardner et al., 2002). A striking biased composition of its DNA was observed, with an overall AT content of 80.6%, a comparable AT enrichment only being observed in the social amoeba *Dictyostelium discoideum* (Eichinger et al., 2005). In *P. falciparum*, AT-rich codons present a significant preference toward encoding asparagines (N) over lysines, explaining why ∼30% of its proteome is rich in long low complexity regions displaying an exceptional abundance of asparagine residues (Aravind et al., 2003; Singh et al., 2004).

Glutamine (Q)- and asparagine (N)-rich sequences have been shown to increase the propensity of a protein to form amyloids, and indeed the expansion of CAG trinucleotide repeats, encoding for Q, in different human proteins, results in developmental and neuromuscular disorders such as Huntington’s disease, Kennedy disease, and several ataxias caused by the accumulation of intracellular protein aggregates in specific neuron types (Orr and Zoghbi, 2007; Williams and Paulson, 2008). Proteins with long N repeats have been shown to have an aggregation propensity even higher than poly-Q stretches (Tartaglia et al., 2005). Intriguingly, in spite of their inherent risk to promote aggregation, sequences with such amino acid compositions are common in yeast prions, and, thus are often referred to as prion domains (PrDs).

Among amyloids, prions are proteins with the unusual ability to adopt different structures and functional states, at least one of which is transmissible between individuals. In yeast, PrDs have been proved to be both sufficient and necessary for prion conformational conversion (Masison and Wickner, 1995). The detailed characterization of the prion phenomenon in yeast has provided important insights on the structural and sequential determinants of PrDs (Alberti et al., 2009). This knowledge has fuelled the development of computational algorithms able to identify prion like domains (PrLDs) in a genome-wide level in other organisms (Michelitsch and Weissman, 2000; Harrison and Gerstein, 2003; Espinosa Angarica et al., 2013, 2014; Lancaster et al., 2014; Zambrano et al., 2015), highlighting the existence of proteins bearing such intriguing sequences in all kingdoms of life (Espinosa Angarica et al., 2013, 2014).

It is now clear that evolution purges out proteins containing aggregation-prone regions, unless these sequences are needed for functional purposes (Monsellier and Chiti, 2007; Chen and Dokholyan, 2008). Given the intrinsic amyloid potential of PrLDs, their biological persistence suggests an evolutionary determination to maintain these regions. The word prion is usually associated with neurodegenerative diseases. However, the recent identification of protein prion-like states executing physiological functions is rapidly changing this notation (Si, 2015). In higher eukaryotes, the earliest examples of functional prion-like polypeptides were described in *Aplysia* and *Drosophila*, where members of the CPEB protein family undergo prion conversion that facilitates memory formation (Heinrich and Lindquist, 2011; Majumdar et al., 2012). Cai et al. (2014) have revealed that the human proteins MAVS and ASC propagate respective downstream signals through prion conversion, and that this signal amplification strategy is crucial for the initiation of the innate immune response. More recently, non-pathogenic prion-like proteins have been described in plants and bacteria: luminidependens, an *Arabidopsis* protein, involved in flowering and plant memory regulation (Chakrabortee et al., 2016) and the transcription terminator Rho factor of the *Clostridium botulinum* pathogen (Pallarès et al., 2016; Yuan and Hochschild, 2017), respectively. These findings suggest that the conformational conversion and subsequent self-assembly that characterize prion-like proteins might be indeed an evolutionary conserved phenomenon (Maji et al., 2009; Tariq et al., 2013; Pallares and Ventura, 2017).

The enrichment of *P. falciparum* in N-rich low complexity sequences, soon suggested that this organism might contain a significant number of prion-like proteins, whose identification might contribute to understand its particular biology (Singh et al., 2004). Bioinformatic analysis found a correlation between the over-representation of homorepeat containing proteins and the abundance of proteins with putative PrLDs, which were proposed to account for as much as 25% of the parasite proteome (Singh et al., 2004). The biological significance of these protein domains is not clear (Muralidharan and Goldberg, 2013), but *P. falciparum* has evolved a very efficient proteostatic system to cope with such an aggregation-prone proteome (Muralidharan et al., 2012; Muralidharan and Goldberg, 2013; Przyborski et al., 2015).

In order to address the potential biological role for prion-like proteins in *P. falciparum*, we analyzed its proteome using a highly stringent computational approach, searching for the presence of Q/N-rich long regions displaying compositional similitude to *bona fide* prions (Toombs et al., 2010, 2012) and bearing specific amyloidogenic regions able to promote their self-assembly (Sabate et al., 2015b; Pallarès et al., 2016; Fernandez et al., 2017). This is the same approach that recently allowed us to propose the transcription terminator Rho factor as a first prion-like protein in bacteria (Pallarès et al., 2016; Pallares and Ventura, 2017). Applying this strategy, we have identified 503 PrLDs-containing proteins in *P. falciparum*, accounting for ∼10% of its proteome. An analysis of the gene ontology (GO) terms enriched in this subproteome indicates that the proteins it contains might participate in important biological processes, such as regulation of gene expression or vesicle-mediated transport. Several of the functions assigned to the putative *P. falciparum* prion-like proteins are common to those reported in other eukaryotes, including humans, while other appear to be specific for this protozoan parasite.

Among all the identified prion-like candidates, we have selected three unrelated proteins and experimentally validated that their PrLDs contain specific short N-rich sequences able to form amyloid fibrils; having thus the potential to trigger the conformational conversion of the *P. falciparum* proteins in which they are embedded.

Collectively, our study suggest that prion-like proteins may play a functional role in the complex parasite’s biology.

## Materials and Methods

### Dataset Construction

The *P. falciparum* (isolate 3D7) reference proteome (Proteome ID UP000001450, released 2015_04, published on April 1, 2015) consisting of 5353 proteins was downloaded from UniProt (UniProt Consortium, 2015). This was first screened for the presence of Q/N-rich domains using an in-house developed Python script. Briefly, it scans for consecutive 80-residue windows and retrieves those with at least 30 Q/Ns. Once applied to *P. falciparum*’s proteome, it rendered 1300 proteins with at least one Q/N-rich domain. These were further scanned with PAPA (using default parameters) for intrinsically disordered regions and compositional bias resembling yeast prions, rendering 581 proteins. A final scan in search for soft amyloid cores within these PrLDs was performed using pWALTZ (using default parameters), resulting in a prion-like dataset of 503 proteins.

### Functional and Structural Enrichment Analysis

Gene ontology (Gene Ontology Consortium, 2015) terms (at the GO FAT category) and Pfam domain enrichment (Finn et al., 2016) were analyzed and clustered with the Functional Annotation Tool of DAVID 6.7 (Database for Annotation, Visualization and Integrated Discovery) (Huang da et al., 2009). The GO term clustering was performed with a high classification stringency and a *p*-value ≤0.05. The Pfam list was obtained with a *p*-value ≤0.05 and final clustering was manually curated. From the 503 proteins in the prion-like proteins dataset, 487 were identified and processed by DAVID. The translation rates of the 10% highest scoring prion-like proteins at the different stages of *P. falciparum* life cycle were retrieved from Le Roch et al. (2003). For every protein entry, the developmental stage with the highest translation rate was considered.

### Peptide Prediction, Synthesis, and Preparation

The sequences of Sec24 (C0H489), the translation initiation factor-like protein IF2c (Q8IBA3) and the protein kinase PK4 (C6KTB8) were also analyzed with the PLAAC (Lancaster et al., 2014) algorithm. The resulting sequences, their position in the full-length protein and their scores are shown in **Figure 2**. The 21-residue peptides corresponding to the PrLDs predicted by pWALTZ were purchased from CASLO ApS (Scion Denmark Technical University). Peptide stock solutions were prepared solubilizing the lyophilized peptides at a final concentration of 5 mM in 100% dimethyl sulfoxide and stored at -80°C. Before each analysis, the samples were diluted to 150 μM in phosphate-buffered saline (PBS) buffer. For aggregation assays, the diluted samples were incubated for 48 h at 25°C.

### Binding to Amyloid Dyes

The fluorescence spectra of the binding of 25 μM Thioflavin-T (ThT) to peptide fibrils were recorded using a Cary Eclipse spectrofluorometer (Varian, Palo Alto, CA, United States) with an excitation wavelength of 440 nm and emission range from 460 to 600 nm at 25°C in PBS buffer. Peptides were equilibrated at room temperature for 2 min before the measurement and solutions without peptide were employed as negative controls. Excitation and emission slit widths of 10 nm were used. For the Thioflavin-S (ThS) staining assays, aggregated peptides were incubated for 1 h in the presence of 125 μM ThS in PBS. Then, the samples were centrifuged (14,000 ×*g* for 5 min) and the precipitated fraction washed twice with PBS and finally placed on a microscope slide and sealed. Images of the aggregated peptides bound to ThS were obtained at 40-fold magnification under UV light or phase contrast in a Leica fluorescence microscope (Leica DMRB, Heidelberg, Germany).

### Secondary Structure Determination

Attenuated total reflectance Fourier transform infrared (ATR FT-IR) spectroscopy analysis of peptide fibrils were performed using a Bruker Tensor FT-IR Spectrometer (Bruker Optics, Berlin, Germany) with a Golden Gate MKII ATR accessory. Each spectrum consisted of 16 independent scans, measured at spectral resolution of 1 cm^-1^ within the 1800–1500 cm^-1^ range. All spectral data were acquired and normalized using the OPUS MIR Tensor 27 software. Infrared spectra between 1725 and 1575 cm^-1^ were fitted through overlapping Gaussian curves, and the amplitude and area for each Gaussian function were calculated employing the non-linear peak-fitting program (PeakFit package, Systat Software, San Jose, CA, United States). Aggregated peptides were prepared at 150 μM in PBS buffer and incubated for 48 h at 25°C. The PBS buffer without peptide was used as a control and subtracted from the absorbance signal before deconvolution.

### Transmission Electron Microscopy

Samples of aggregated peptides obtained as described previously, were placed onto carbon-coated copper grids and incubated for 5 min. The grids were washed with distilled water and negatively stained with 2% (w/v) uranyl acetate for 2 min. Micrographs were obtained in a JEM-1400 (JEOL, Japan) transmission electron microscope (TEM) operated at 80 kV accelerating voltage.

### *In vivo* Amyloid-Like Detection

Cultures of *P. falciparum* strain 3D7 were grown *in vitro* in group B human red blood cells (RBCs), purchased from the Banc de Sang i Teixits^1^, using previously described conditions (Cranmer et al., 1997). Briefly, parasites (thawed from glycerol stocks) were cultured at 37°C in T-Flasks containing RBCs in Roswell Park Memorial Institute (RPMI) complete medium under a gas mixture of 92% N_2_, 5% CO_2_, and 3% O_2_. Synchronized cultures were obtained by 5% sorbitol lysis (Lambros and Vanderberg, 1979) and the medium was changed every 2 days maintaining 3% hematocrit and a parasitemia below 5%. Staining with PROTEOSTAT^®^ protein aggregation assay (Enzo Life Sciences, Inc.) was performed according to the manufacturer’s instructions. Briefly, 200 μl of *P. falciparum* culture were harvested and washed twice with 1 ml of 7.5 mg BSA/ml PBS (PBS/BSA); the resulting cell pellet was taken up in 200 μl of PBS/BSA containing 2 μg/ml Hoechst 33342 and PROTEOSTAT^®^ (1:3000 stock dilution), and incubated for 30 min at room temperature in the dark before being washed again twice with 1 ml of PBS/BSA. 10 μl of the washed cell suspension were transferred into a Lab-Tek chambered coverglass (Nunc, Thermo Fisher Scientific) containing 180 μl of PBS/BSA and finally analyzed with a Leica TCS SP5 laser scanning confocal microscope, using a 63× immersion oil objective with 1.4 numeric aperture. Hoechst 33342 and PROTEOSTAT^®^ were detected, respectively, by excitation through 405 and 488 nm lasers. Emission was collected between 415 and 500 nm for Hoechst 33342, and between 590 and 670 for PROTEOSTAT^®^.

## Results

### The *P. falciparum* Proteome Is Enriched in Proteins With PrLDs

The *P. falciparum* proteome contains an unusually high amount of low complexity regions; long domains enriched in certain amino acids and without a defined secondary structure. Low complexity regions are present in 30–90% of *P. falciparum* proteins, depending on the detection stringency (Singh et al., 2004; DePristo et al., 2006), and they are specially enriched in N residues. It has been proposed that these disordered protein regions might share certain properties with the classical yeast Q/N-rich PrDs (Faux et al., 2005; Tompa and Fuxreiter, 2008; Fuxreiter, 2012; Fuxreiter and Tompa, 2012; Malinovska et al., 2013; Pallarès et al., 2016), and potentially support the formation of prion-like macromolecular assemblies (Singh et al., 2004; Espinosa Angarica et al., 2013).

In order to evaluate the presence of Q/N-rich prion-like proteins in *P. falciparum*, we examined its proteome combining detection of local Q/N-enrichment together with PAPA and pWALTZ predictions (Toombs et al., 2012; Zambrano et al., 2015; Pallarès et al., 2016) (see section “Materials and Methods”). Thus, any predicted PrLD in our subproteome would fulfill the following requirements: being Q/N-rich, disordered (PAPA includes the disorder predictor FoldIndex; Prilusky et al., 2005), compositionally similar to yeast PrDs and contain a short sequence stretch able to facilitate its conversion into an amyloid-like state; we have generically named these stretches “soft amyloid cores,” because their amyloid propensity is significantly lower than the classical amyloid regions of pathogenic proteins, but still enough to promote protein self-assembly (Batlle et al., 2017).

The analysis shows that 1300 proteins (24.3% of the proteome) bear at least one Q/N-rich domain, in excellent agreement with previous studies (Singh et al., 2004). 581 of these Q/N-rich domains (44.7%) also display an amino acid composition similar to that of yeast PrDs and are disordered, as predicted by PAPA, and among them, 503 domains (86.6%) contain a soft amyloid core as predicted by pWALTZ (**Supplementary Table S1**). Overall, we conclude that 9.4% of the *P. falciparum* proteome may have the physicochemical properties and the aggregation potential to behave as a prion. This value is lower than previously estimated applying other computational approaches based only in Q/N richness (Singh et al., 2004) or than the one estimated using only compositional similitude to yeast PrDs with PAPA, which predicts 22.5% of the parasite proteins as prion-like. The Q/N rich only dataset contains many long poly-N stretches, without any inner hydrophobic residue, a requisite to act as a prion (Toombs et al., 2010), whereas the PAPA only dataset include polypeptides unlike to behave as prions *in vivo*, like membrane integral proteins, only because they display sequence stretches enriched in certain hydrophobic residues. In any case, even with the stringent approach used here, roughly 10% of the *P. falciparum* proteome seems to correspond to proteins displaying PrLDs and may thus have a high intrinsic aggregation propensity. This generates several important questions: Why *P. falciparum* proteins are so rich in PrLDs? Which are these putative prions? What are their roles in *P. falciparum*?

Previous works addressed these questions by analyzing the distribution of Q/N- or N-rich regions in the *P. falciparum* proteome (Singh et al., 2004). They detected such stretches in all protein families and all developmental stages of *P. falciparum*, without an evident association with any specific biological process. However, an increasing number of studies are uncovering PrLDs being connected to specific functions and processes in other species (Espinosa Angarica et al., 2014; Iglesias et al., 2015). We hypothesized that focusing the analysis in our curated subproteome may help to unravel the functional purpose, if any, of PrLDs in *P. falciparum*.

### Computational Analysis of the Role of Prion-Like Proteins in *P. falciparum*

The DAVID Functional Annotation Clustering Tool was employed to identify enriched GO categories (Huang da et al., 2009; Gene Ontology Consortium, 2015) in the previously identified *P. falciparum* PrLD-containing proteins (*p*-value ≤ 0.05). It is worth to mention that this analysis is constrained by the fact that 60% of *P. falciparum* genes have unknown functions (Gardner et al., 2002), most of them have no clear homolog in other eukaryotes, and that the mechanisms underlying the main processes related to malaria pathogenesis in *P. falciparum* are still poorly understood. However, 487 out of the 503 proteins in our dataset were identified and processed by DAVID, which results in a coverage of 94.8% of our subproteome.

**FIGURE 1:**
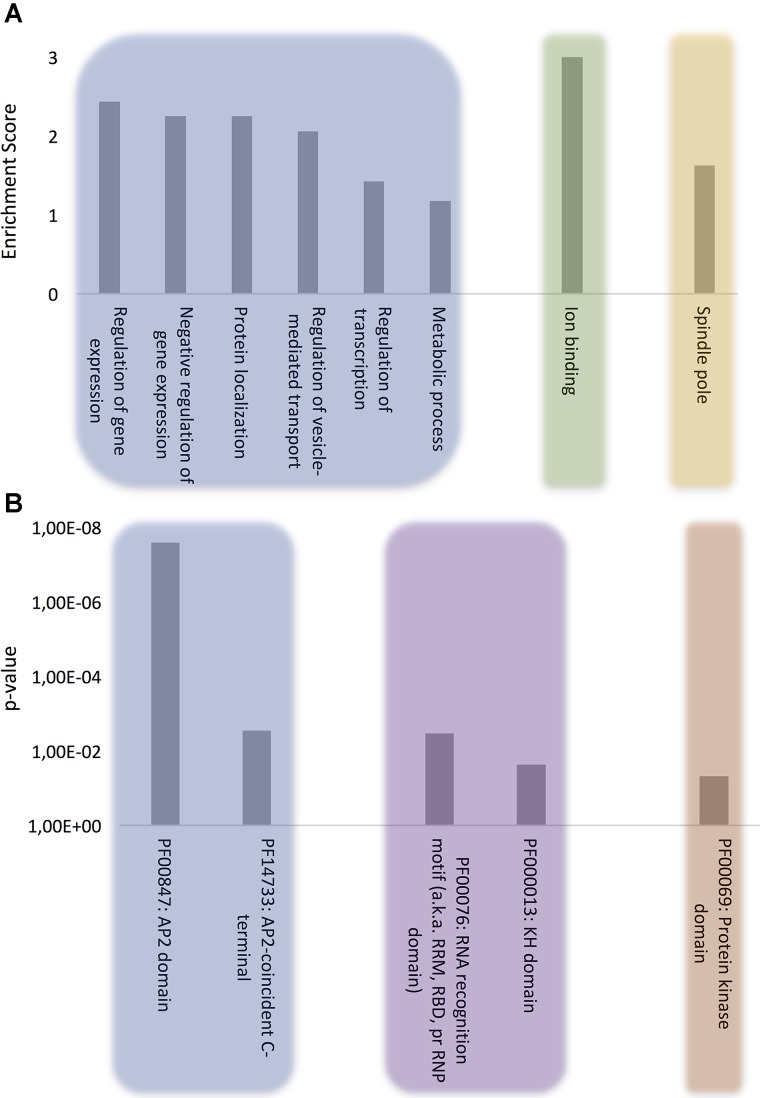
Computational analysis of the role of *P. falciparum* PrLD-containing proteins. **(A)** GO biological process, molecular function, and cellular component terms enriched in *P. falciparum* PrLD-containing proteins. **(B)** Pfam structural domains enriched in PrLD-containing proteins. The enrichment analysis was performed with Functional Annotation Tool of DAVID 6.7 using high stringency, *p*-value ≤0.05 for GO and Pfam terms.

The proteins were clustered according to the following ontologies: biological process, molecular function, and cellular component (**Figure 1A** and **Supplementary Table S2**). The most significant biological process gene clusters include “regulation of gene expression,” “negative regulation of gene expression,” and “regulation of transcription.” The abundance of DNA and RNA binding proteins with PrLDs (**Figure 1B**) is consistent with the observation that many of the prion-like proteins discovered initially in yeast (Alberti et al., 2009) and more recently in humans (King et al., 2012), plants (Chakrabortee et al., 2016) and bacteria proteomes (Iglesias et al., 2015) are proteins associated with gene expression and translation regulation such as transcription factors and RNA-binding proteins.

Other biological process terms could also be arranged into enriched clusters: “protein localization,” “regulation of vesicle-mediated transport,” and “metabolic process.” Importantly, the vesicle-mediated transport system and the trafficking of parasite proteins to diverse locations in the host cell are essential to promote new parasite phenotypes, playing a crucial role in host-pathogen interactions, as well as in disease pathogenesis and susceptibility (Miller et al., 2002; Hiller et al., 2004; Marti et al., 2005). Indeed, extracellular vesicles have been shown to act as delivery agents for prion-like proteins (Liu et al., 2017).

The analysis of molecular function domains in the set of *P. falciparum* prion-like proteins revealed that the only significantly enriched cluster was ion binding. A deeper analysis of the GO annotations indicates that ∼33% of these proteins function in DNA/RNA interaction and ∼40% of them also contain structural domains related to nucleotide binding, such as Zinc fingers. In fact, the functions associated to nucleotide binding, especially RNA binding, appear to be associated to proteins containing PrLDs, regardless of the organism (Espinosa Angarica et al., 2014; Iglesias et al., 2015; Pallarès et al., 2016).

At this point, to dig a bit more on the functional role of our protein subset, we reanalyzed the MF category setting a *p*-value cutoff of 0.1. Several new GO terms came to light that could be grouped into two interesting MF subclusters: (i) chromatin remodeling, which is consistent with recent studies demonstrating that the physical properties of PrLDs can retarget critical chromatin regulatory complexes (Boulay et al., 2017) and facilitate heterochromatin assembly (Kataoka and Mochizuki, 2017) and (ii) GTPase regulatory activity, which is also detected in the PrLD-containing proteins of several other organisms (bacteria, plants, fungi, and invertebrates) (Espinosa Angarica et al., 2013); indeed, the canonical and best characterized yeast prion, Sup35, is a GTPase (Glover et al., 1997).

Analysis of the cellular component ontology category shows a specific enrichment at the spindle pole. In yeast, prion proteins have been shown to interact specifically with spindle pole proteins (Treusch and Lindquist, 2012) and spindle-associated proteins have been shown to be involved in self-assembly mediated phase separation in Xenopus (Jiang et al., 2015).

All the proteins in our Q/N-rich dataset have in common the presence of a disordered region of 80 amino acids in which at least the 37.5% of the residues (30/80) correspond to Q or N. This compositional similitude might imply a certain overlap of functions between proteins bearing PrLDs and those devoid of them. A GO analysis of Q/N-rich proteins without PrLDs (**Supplementary Figure S1**), shows that the molecular function and cellular location terms are different, but related, to those found in the Q/N-rich protein subset bearing PrLDs; nucleotide binding and cytoskeleton being the most enriched terms for these two categories, respectively. In contrast, Q/N-rich proteins without PrLDs are poorly represented in specific biological processes, being the most enriched one DNA repair (**Supplementary Figure S1**). This suggests that the compositional/sequential features of PrLDs might be important to specify the biological context in which the proteins act, whereas their generic molecular function depends mostly on the local enrichment in Q/N residues.

Prion-like proteins display a modular architecture in which one or several long and disordered PrLDs are adjacent to conventional globular domains and, accordingly, they tend to be large. We compared the average size of our protein subset with the one of the complete plasmodium proteome, confirming that proteins bearing PrLDs are effectively significantly longer (**Supplementary Figure S2**). To discard that the GO terms identified for PrLD-containing proteins would respond only to their differential size, we selected the subset of the largest 503 proteins in the proteome and performed a GO analysis. The resulting enriched terms did not coincide with those in our subset in any of the categories. The most enriched biological processes in large proteins were pathogenesis and single organismal cell–cell adhesion; the most enriched compartments were infected host cell surface knob and host cell plasma membrane and the most enriched molecular functions were receptor activity and cell adhesion molecular binding.

In order to address if the expression of PrLD-containing proteins occurs preferentially at a given parasite stage, we analyzed the expression levels of the 10% top ranking proteins in our dataset at each of the different life cycle stages, as reported by Winzeler and colleagues (Le Roch et al., 2003). It turns out that, on the average, the highest translation rates for these proteins correspond to those at the merozoite and early ring stages (**Supplementary Figure S3**).

### Protein Domains in *P. falciparum* Prion-Like Proteins

To further evaluate the role of our collection of PrLD-containing proteins, we examined in detail their constituent functional domains (Finn et al., 2016) (**Figure 1B**). As expected, after clustering, the Pfam domains that were most often found in combination with PrLDs were involved in DNA/RNA binding, among which, the ApiAP2 stands out. The ApiAP2 family is homologous to the plant Apetala2/ethylene response factor (AP2/ERF) DNA-binding proteins, which comprise the second largest class of transcription factors in *A. thaliana*. Balaji et al. (2005) described that ApiAP2 proteins are likely to function as a family of apicomplexan parasite-specific transcription factors and that their amino acid sequences are highly conserved among orthologs. Strikingly, our data reveals that at least the 50% of the members composing this family in *P. falciparum* contain a PrLD. Several studies support their major role in mediating the regulation of stage-specific gene expression profiles in the parasite’s development (Yuda et al., 2010; Painter et al., 2011; Modrzynska et al., 2017) and suggest their crucial contribution to *P. falciparum* complexity and growth since very few ApiAP2 genes have been successfully knocked out (Behnke et al., 2010; Yuda et al., 2010).

The RNA recognition motif (RRM) is the most enriched RNA-binding domain (RBD) in our dataset. RRMs are by far the most versatile and abundant RBDs, their fold being conserved from bacteria to higher eukaryotes (Reddy et al., 2015). This result is consistent with the observation that many of the human proteins with PrLDs contain an RRM motif and are involved in liquid–liquid phase transitions facilitating the formation of dynamic membraneless intracellular compartments, such as ribonucleoprotein (RNP) granules. They allow material exchange and fast assemblage and adaptation to different environments and cell states (Malinovska et al., 2013), the PrLDs in RNA binding proteins provide the special physicochemical properties that allow contacts between RNAs and proteins that sustain the liquid-like assemblies (Han et al., 2012; Kato et al., 2012). Indeed, the second most abundant RBD linked to our protein subproteome is the KH domain, a protein domain that was first identified in the human heterogeneous nuclear proteins (Siomi et al., 1993) and, together with RRM, constitutes the most abundant domain in RNA granules forming proteins (Kato et al., 2012). In *P. falciparum* RNPs are involved in translation repression and posttranscriptional regulation of gene expression, critical for some stages of the parasite (Kramer, 2014).

The last enriched Pfam family includes the protein kinase domain. It is well-known that phosphorylation/dephosphorylation is the major control mechanism for many cellular functions. Consistently, recent studies carried out in *P. falciparum* reveal stage-specific profiles of protein phosphorylation, suggesting that reversible protein phosphorylation plays a key role in the regulation of the *Plasmodium* life cycle (Wu et al., 2009; Pease et al., 2013). So far, no PrLD-containing protein kinase has been characterized experimentally, but it is obvious that the ability to control the activity of these enzymes by modulating their assembly would have important physiological consequences.

### Predicted PrLD Soft Amyloid Cores in *P. falciparum* Proteins

Based on the above computational results, we selected three PrLD-containing proteins for their experimental characterization: the putative transport protein Sec24 (C0H489), the translation initiation factor-like protein IF2c (Q8IBA3), and the protein kinase PK4 (C6KTB8). These proteins are associated with functions (nucleotide binding, Q8IBA3), cellular components (vesicle mediated-transport, C0H489) and structural domains (kinase, C6KTB8) that are enriched in our dataset. The selected candidates have no functional or sequential relationship and have not been previously suggested to act as prions.

As a first candidate, we chose Sec24b, a member of the Sec24 family. Within this family, Sec24b is by far the most enriched in Q/N, which constitute 21.5% of the amino acids in the complete sequence and 51.9% of the PrLD. Sec24b is closely related to the mammalian Sec24C/D family and the yeast Sec24 homolog Lst1 (Lee et al., 2008). These proteins play a key role in shaping the vesicle, as well as in cargo selection and concentration (Roberg et al., 1999). They have a scaffolding function required to generate vesicles that can accommodate difficult cargo proteins, including large oligomeric assemblies.

As a second candidate, we selected IF2, one of the essential components for the initiation of protein synthesis. IF2 is a translation initiator factor acting as a GTPase that recruits the charged fMet-initiator tRNA onto the 30S ribosomal initiation complex (Antoun et al., 2003). From the three IF2 homologs described in *P. falciparum* (Haider et al., 2015), IF2c is the only one that holds a PrLD. It is worth to note that the IF2c C-terminal domain, where the PrLD maps, has the largest identity with bacterial IF2, a family of translation initiation factors rich in putative PrLDs (Iglesias et al., 2015). 20.8% of IF2c residues are Q/N and this proportion raises to 49.4% in the PrLD.

The third selected protein was the kinase PK4, a protein that is essential for completion of the blood stage of the disease (Zhang et al., 2012). Thus PK4 has been suggested as a novel target for the next generation of antimalarial compounds (Kahrstrom, 2012). PK4 phosphorylates eIF2α and arrests global protein synthesis in schizonts (mature form of the blood cycle) and gametocytes (sexual form that infects the mosquitoes). 17.7% of PK4 amino acids are Q or N, with 55.5% of its PrLD corresponding to these polar residues.

**FIGURE 2:**
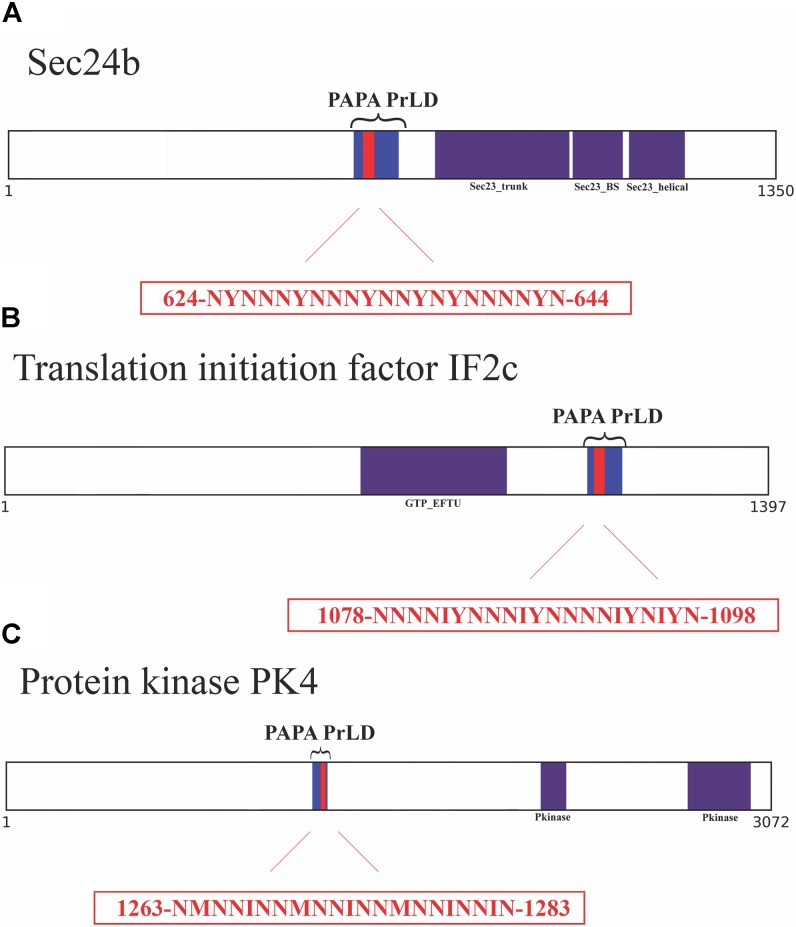
Soft amyloid cores prediction in the three candidate proteins. **(A)** Sec24b diagram showing the location of the PrLD predicted by PLAAC (blue, position 608–686) or PAPA (brackets, position 607–687). **(B)** IF2c location of the PrLD predicted by PLAAC (blue, position 1066–1134) or PAPA (brackets, position 1057–1137). **(C)** PK4 location of the PrLD predicted by PLAAC (blue, position 1230–1290) or PAPA (brackets, position 1220–1300). Pfam domains and the amyloid cores are shown in purple and red, respectively, the exact position and the sequence of the amyloid cores are presented in the red box.

To further confirm the presence of PrLDs in these proteins, and to define more precisely their boundaries, we used PLAAC (Lancaster et al., 2014), yet another composition-based predictor, in which, in contrast to PAPA, the length of the predicted PrLD also depends on the protein composition. PLAAC detected PrLDs overlapping with the regions previously identified by PAPA, in the three polypeptides (**Figure 2**).

We analyzed these three putative prion-like proteins using the same computational approach, we employed previously to detect and validate the soft amyloid cores present in *bona fide* yeast prions (Sant’Anna et al., 2016), in the pathogenic bacteria *C. botulinum* (Pallarès et al., 2016) and in human prion-like proteins (Batlle et al., 2017). The predicted soft amyloid cores for these *P. falciparum* proteins are shown in **Table 1**. Not surprisingly, these stretches are highly enriched in N residues, all containing >60% of this polar residue. Interestingly enough, well-validated aggregation predictors like Aggrescan, Tango, and Zyggregator (Fernandez-Escamilla et al., 2004; Conchillo-Sole et al., 2007; Tartaglia and Vendruscolo, 2008), all failed to classify these stretches as aggregation-prone (**Supplementary Table S3**), likely because of their much lower hydrophobicity, when compared with the classical amyloid stretches present in pathogenic amyloidogenic proteins (**Supplementary Table S4**). One of the restrains in our prediction scheme is that the identified PrLD should be essentially disordered, as predicted with FoldIndex (Prilusky et al., 2005). In this structural context, the identified soft amyloid cores will be mostly exposed to solvent and able to establish intermolecular contacts, if they have this ability. Orthogonal analysis with alternative disorder prediction algorithms confirms that this is likely the case for the three proteins herein (**Supplementary Table S5**).

**Table 1:** Predicted PrLD soft amyloid cores.

Protein	UniProt ID	PrLD amyloid core	*N* (%)	pWALTZ score	PAPA score	PLAAC score
Sec24b	C0H489	624-NYNNNYNNNYNNYNYNNNNYN-644	71	84.62	0.20	45.28
IF2c	Q8IBA3	1078-NNNNIYNNNIYNNNNIYNIYN-1098	62	87.71	0.07	36.18
PK4	C6KTB8	1263-NMNNINNMNNINNMNNINNIN-1283	67	77.34	0.25	47.97


*For each PrLD-containing protein, it is shown the UniProt ID, the 21 residue-long soft amyloid core with its respective position in the sequence, pWALTZ score, PAPA score, and PLAAC score with a cutoff of 73.55, 0.05, and >0, respectively. PAPA and PLAAC search for compositional similarity to yeast prion domains, defining the predicted PrLD, while pWALTZ scans for soft amyloid cores within them*.

We synthesized 21-residue-long peptides corresponding to the detected soft amyloid cores and characterized their amyloid properties experimentally.

### Predicted PrLDs Soft Amyloid Cores Assemble Into β-Sheet Rich Structures

**FIGURE 3:**
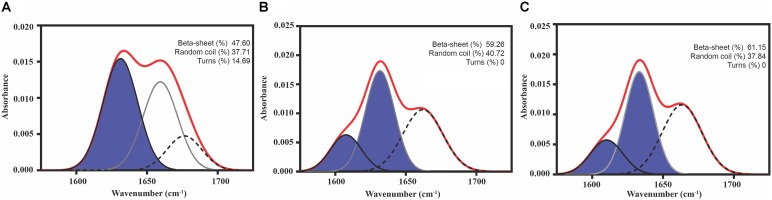
Predicted PrLD Soft amyloid cores secondary structure. Secondary structure determined by ATR FT-IR in the amide I region. The red line corresponds to the absorbance spectrum; the blue area indicates the contribution of the inter-molecular β-sheet signal to the total area upon Gaussian deconvolution. **(A)** Sec24b, **(B)** IF2c, and **(C)** PK4.

As a first evaluation of the assembling properties of the selected peptides, we measured their ability to adopt a β-sheet-enriched structure, a hallmark of amyloid fibril formation (Nelson et al., 2005). To this aim, the peptides were prepared at 150 μM in PBS and incubated during 48 h at 25°C. We used FT-IR spectroscopy and recorded the amide I region of the spectrum (1700–1600 cm^-1^) (**Figure 3** and **Supplementary Table S6**). This spectral region corresponds to the absorption of the carbonyl peptide bond group of the protein main chain and is sensitive to the protein conformation. Deconvolution of the spectra allowed us to assign the secondary structure elements and their relative contribution to the main absorbance. In the three cases, the main peaks mapped in the 1620–1630 cm^-1^ region of the spectra, accounting for 50% or more of the absorbance signals, indicating that the peptides have acquired significant intermolecular β-sheet structure. Interestingly, no anti-parallel β-sheet band was detected (∼1690 cm^-1^) in any of the samples; thus, suggesting that the detected β-strands in the self-assembled peptides would adopt preferentially a parallel disposition. The other detected signals were associated with the presence of disordered structure and turns (**Figure 3** and **Supplementary Table S6**).

### Predicted PrLD Soft Amyloid Cores Form Amyloid-Like Fibrillar Structures

**FIGURE 4:**
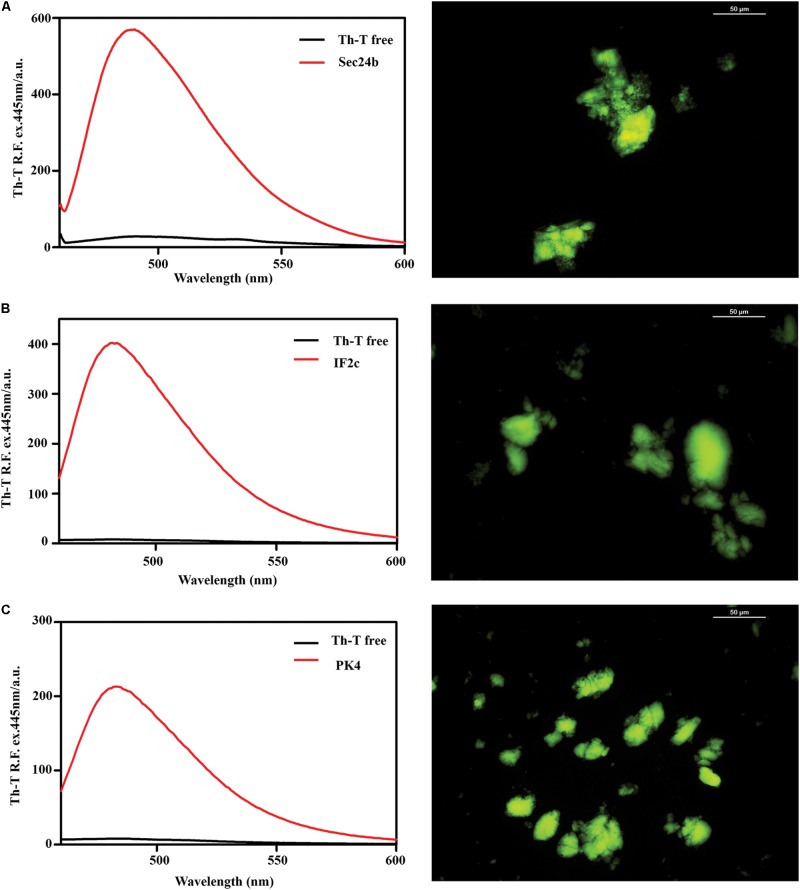
Binding of the predicted PrLD soft amyloid cores to specific amyloid dyes. Fluorescence emission spectrum of ThT when excited at 440 nm; note the characteristic fluorescence enhancement at ∼480 nm when the dye is bound to amyloid-like aggregates. On the right side of the panel, ThS binding of aggregated peptides at 150 μM in PBS after 48 h of incubation at 25°C. The typical green fluorescence can be observed under the fluorescence microscope, images were obtained at 40× magnification. **(A)** Sec24b, **(B)** IF2c, and **(C)** PK4.

To assess if the identified β-sheet-rich assemblies correspond to amyloid-like structures, we used the amyloid-specific dyes ThT and ThS. After incubation at a concentration of 150 μM in PBS during 48 h at 25°C, all the peptides were able to promote a large increase in the intensity of ThT fluorescence emission (**Figure 4**). In addition, areas rich in fibrous material were stained by ThS to yield a bright green-yellow fluorescence against a dark background (**Figure 4**).

**FIGURE 5:**
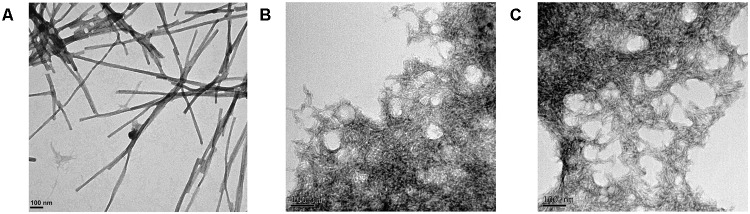
Fibrillar structures formed by the predicted PrLD soft amyloid cores. Representative TEM images for **(A)** Sec24b, **(B)** IF2c, and **(C)** PK4 aggregated peptides at 150 μM in PBS after 48 h of incubation at 25°C.

Transmission electron microscopy examination of the morphological features of the incubated peptide solutions (**Figure 5**) revealed that they effectively assemble into supramolecular structures. Sec24b formed long and straight fibrils, whereas IF2c and PK4 formed short and curly fibrillar structures.

Overall, biophysical analysis of the three predicted peptides demonstrates the ability of the candidate *P. falciparum* soft amyloid cores to nucleate the formation of β-sheet-rich amyloid-like structures.

### Detection of Intracellular Protein Aggregates in *P. falciparum*

The above experimental data suggest that ∼10% of the *P. falciparum* proteome might possess the ability to establish amyloid-like contacts, at least transiently, *in vivo*, and thus; that at any time, a significant number of proteins might potentially aggregate in the parasite. We employed a permeable amyloid-specific dye (PROTEOSTAT^®^) to track the *in vivo* presence of intracellular amyloid-like aggregates in *P. falciparum*.

**FIGURE 6:**
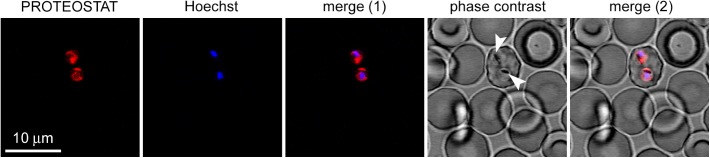
Fluorescence microscopy analysis of the presence of protein aggregates in *P. falciparum-*infected RBCs (pRBCs). The selected field shows a single pRBC in early trophozoite stage, indicated by its characteristic nuclear Hoechst blue fluorescence among enucleated non-parasitized erythrocytes. The amyloid-specific dye PROTEOSTAT^®^ reveals protein aggregates in the cytosol of the two parasite cells present in the pRBC. The arrowheads indicate nascent hemozoin crystals in the food vacuole of *Plasmodium*.

*Plasmodium falciparum* was grown in RBCs and then the culture was incubated with the amyloid dye. We observed colocalization between PROTEOSTAT^®^ fluorescence and the cytosol of *P. falciparum*-infected RBCs, whose nuclei stained with Hoechst 33342. The images evidenced the lack of structures able to bind the dye in non-infected erythrocytes, and that, accordingly, only upon infection by *Plasmodium*, red fluorescent amyloid foci are evident inside parasitized RBCs (**Figure 6**), demonstrating the high amyloid load that this parasite supports at this stage.

## Discussion

Many lines of evidence suggest that prion-like proteins can be both harmful and beneficial for the cell. The propensity of a protein to behave as a prion is encoded in its amino acid sequence (Sabate et al., 2015a). In particular, long and disordered N/Q-rich sequences seem to facilitate conformational conversion into functional amyloid-like states (Alberti et al., 2009). The occurrence of N/Q-rich sequence stretches varies substantially between organisms, with *P. falciparum* having one of the most enriched proteomes in this kind of regions, and specifically in N-rich sequences (Aravind et al., 2003). Accordingly, it has been assumed that prion-like proteins would be common in this organism (Michelitsch and Weissman, 2000). Long Q- and N-homorepeats are inherently aggregation-prone (Halfmann et al., 2011). However, these sequence stretches alone are not sufficient to sustain a prion-like behavior (Toombs et al., 2010). Here, using a stringent computational approach that considers that PrLDs should not be only Q/N-rich, but also display compositional similitude to *bona fide* yeast PrDs and encode for at least one specific short sequence stretch with moderate, but significant, amyloid propensity (Sabate et al., 2015b), we concluded that 503 polypeptides in *P. falciparum* fulfill the requirements to potentially behave as prion-like proteins. This accounts for ∼10% of the proteome, which despite being a lower fraction than previously proposed (∼25%) (Singh et al., 2004), still might constitute a high prionic load for the parasite.

*A priori*, the presence of PrLDs might be dangerous for *Plasmodium*, since prion-like proteins have an intrinsic propensity to aggregate and, in humans, disease-linked mutations occur preferentially in the PrLDs of these polypeptides (Kim et al., 2013). On the other hand, these PrLDs might act as conformational switches that control protein assembly and thus protein function to allow adaptation to the changing environment that *P. falciparum* faces during its life cycle. Importantly, the PrLD-containing subproteome, we identify here is associated with defined domains and functionalities in the parasite, which suggests that Q/N-rich PrLDs do not occur randomly in the *P. falciparum* proteome. This assumption is supported by the fact that PrLDs are associated with similar GO-clusters in organisms as divergent as *Plasmodium*, yeast, *Dictyostelium*, *Drosophila* and humans (Malinovska et al., 2013, 2015). For instance, the role of PrLDs-containing proteins in DNA and RNA binding is well conserved, with the RRM domain being among the most enriched PrLD-associated domains in these organisms. Indeed, 25% of the *P. falciparum* proteins bearing an RRM domain also contain a predicted PrLD. We found that this domain association is also conserved in *Plasmodium vinckei* and *Plasmodium yoelii*, with 13 and 15% of RRM-containing proteins having a Q/N-rich PrLD.

*Plasmodium falciparum* PrLDs exhibit specific associations with domains and functions not detected in other organisms, such as the ApiAP2 proteins, with 50% of their members displaying a PrLD. These proteins have been postulated as the main transcriptional regulators in *Plasmodium* parasites and the other Apicomplexa (Balaji et al., 2005). Importantly, according to our analysis, the presence of PrLDs within AP2 transcription factors also seems to be evolutionary, with 39, 29, and 24% of the AP2 proteins in *P. vinckei*, *P. yoelii*, and *Plasmodium berghei* displaying Q/N-rich PrLDs, respectively. The association between PrLDs and the regulation of vesicle-mediated transport is also a specific feature of *Plasmodium*. This process allows the trafficking of some parasite proteins to the erythrocyte membrane (Miller et al., 2002; Hiller et al., 2004; Marti et al., 2005).

*Plasmodium* is an obligate parasite that has evolved to survive in different hosts and cell types. It has a complex life cycle with cellular stages that differ in shape, size, metabolic activity, and resource requirements. Hence, to sustain this complexity, *Plasmodium* requires an efficient regulation, to which the conformational conversion of regulatory proteins bearing PrLDs might contribute. Changes in local protein concentration, binding to nucleic acids and posttranslational modifications have been shown to modulate the assembly of PrLDs, the functional outcome depending on the particular assembled protein (Alberti, 2017).

We and others have suggested that certain short amyloidogenic sequence stretches embedded in PrLDs contribute significantly to prion formation, maintenance, and transmission, at least in yeast (Crow et al., 2011; Sabate et al., 2015a; Sant’Anna et al., 2016; Batlle et al., 2017). The computational search for such regions in the putative PrLDs of a large number of bacterial proteomes (Iglesias et al., 2015), previously thought to lack prions, and a subsequent experimental validation, allowed us to propose that the Rho transcription terminator might constitute a first bacterial prion (Pallarès et al., 2016; Pallares and Ventura, 2017). Soon after, Yuan and Hochschild (2017) confirmed the ability of this protein to adopt an infectious state, leading to global changes in the transcriptome. Here, we used the same approach to study the amyloidogenic potential of three *P. falciparum* PrLD-containing proteins: the translation initiation factor 2c, the kinase PK4, both involved in gene expression regulation (Zhang et al., 2012; Haider et al., 2015) and Sec24b, involved in vesicle trafficking (Lee et al., 2008). Our data provide compelling evidence that, *in vitro*, all three candidate proteins contain short nucleating regions embedded in the PrLDs able to spontaneously self-assemble into amyloid-like structures. The presence of such stretches does not necessarily imply that the correspondent large full-length proteins would behave in a prion-like manner, and this behavior should be experimentally validated. However, several indirect evidences suggest that this could be the case: (i) we have shown that when a predicted short amyloid sequence is administered to cells in its amyloid state it is able to seed the conformational conversion of the complete endogenous protein and its subsequent aggregation into a prionic form (Sant’Anna et al., 2016), (ii) the amyloid stretch, we identified in the PrLD of the bacterial Rho terminator factor has been shown to be absolutely essential for its self-assembly and prion activity (Yuan and Hochschild, 2017), and (iii) Vorberg and colleagues have shown that the amyloid sequence, we pinpointed in the PrLD of a model prion protein is the only region required for the induction, propagation, and inheritance of the prion state in the mammalian cytosol (Duernberger et al., 2018).

Overall, we identified a subset of putative Q/N-rich prion-like proteins in *P. falciparum* associated with specific biological processes and experimentally validated that their highly polar and disordered PrLDs contain cryptic sequences able to self-assemble into amyloids. The structural characterization and *in vivo* validation of the properties of the identified proteins is challenging, but it is worth the effort, since it might uncover a first *bona fide* prion in *Plasmodium*.

## Author Contributions

IP, XF-B, and SV conceived the experiments. IP, NG, VI, RS, and AB conducted the experiments. IP, NG, VI, XF-B, and SV analyzed the results. IP, NG, VI, and AB prepared the figures. IP, NG, and SV wrote the main manuscript text. All authors reviewed the manuscript.

## Conflict of Interest Statement

The authors declare that the research was conducted in the absence of any commercial or financial relationships that could be construed as a potential conflict of interest.

## Footnotes

1 http://www.bancsang.net
