# Supplementary material for: Discovering Putative Prion-Like Proteins in Plasmodium falciparum: A Computational and Experimental Analysis
Source: Front Microbiol. 2018 Aug 7;9:1737. doi: 10.3389/fmicb.2018.01737 (PMC6090025; doi:10.3389/fmicb.2018.01737)
Supplement: Supplementary file 3 [file Table_3.pdf]

Table S3. ***P. falciparum*** **PrLD soft amyloid cores aggregation prediction.** Analysis of the aggregation tendencies of the *P. falciparum* soft amyloid cores candidates using Aggrescan (Conchillo-Sole, et al., 2007), Tango (Fernandez-Escamilla, et al., 2004) and Zyggregator (Tartaglia and Vendruscolo, 2008) algorithms. They all failed to predict any significant amyloid propensity in the peptides.

PROTEIN	PrLD AMYLOID CORE	AGGRESCAN	TANGO (%)	ZYGGREGATOR
**Sec24b**	NYNNNYNNNYNNYNYNNNNYN	-44.40	0	-4.14
**IF2c**	NNNNIYNNNIYNNNNIYNIYN	-27.0	2.55	-0.93
**PK4**	NMNNINNMNNINNMNNINNIN	-26.40	18.32	-3.23
