# Supplementary material for: Discovering Putative Prion-Like Proteins in Plasmodium falciparum: A Computational and Experimental Analysis
Source: Front Microbiol. 2018 Aug 7;9:1737. doi: 10.3389/fmicb.2018.01737 (PMC6090025; doi:10.3389/fmicb.2018.01737)
Supplement: Supplementary file 4 [file Table_4.pdf]

**Table S4. *P. falciparum* PrLD soft amyloid cores hydropathicity**. For each candidate, GRAVY score (average hydrophobicity and hydrophilicity) was evaluated using the EXPASY ProtParam tool (Gasteiger, et al., 2003). Positive values corresponding to hydrophobic sequences are shown in bold, negative values correspond to hydrophilic sequences. The pathogenic proteins α-synuclein (ASYN) and Aβ42 short amyloidogenic stretches were predicted with AmylPred2 (Tsolis, et al., 2013).

| PROTEIN | PrLD AMYLOID CORE | GRAVY SCORE |
|---|---|---|
| **Sec24b** | NYNNNYNNNYNNYNYNNNNYN | -2.87 |
| **IF2c** | NNNNIYNNNIYNNNNIYNIYN | -1.56 |
| **PK4** | NMNNINNMNNINNMNNINNIN | -1.21 |
| **ASYN** | GVLYVG | **1.683** |
| | GGAVVTGVTAVAQ | **1.238** |
| **Aβ42** | GAIIGLMVGGVVI | **2.462** |
| | QKLVFFAE | **0.562** |
