# Supplementary material for: Discovering Putative Prion-Like Proteins in Plasmodium falciparum: A Computational and Experimental Analysis
Source: Front Microbiol. 2018 Aug 7;9:1737. doi: 10.3389/fmicb.2018.01737 (PMC6090025; doi:10.3389/fmicb.2018.01737)
Supplement: Supplementary file 5 [file Table_5.pdf]

Table S5. Disorder context of PrLD soft amyloid cores. To predict disorder, FoldIndex (Prilusky, et al., 2005), PONDR-FIT (Xue, et al., 2010), IUPRED (Dosztanyi, et al., 2005), RONN (Yang, et al., 2005) algorithms were used. Disorder was analyzed for the 21 residues-long peptides and 20 flanking residues at each end and expressed as the percentage of disordered residues in these 61 residues-long segments. Average disorder accounts for the mean of all disorder predictions for a given segment.

**DISORDER PREDICTOR**	**Sec24b**	**IF2c**	**PK4**
FoldIndex	100	100	100
PONDR-FIT	100	100	100
IUPRED	79	80	61
RONN	69	62	69
**AVERAGE**	**87**	**86**	**83**
