# Supplementary material for: Discovering Putative Prion-Like Proteins in Plasmodium falciparum: A Computational and Experimental Analysis
Source: Front Microbiol. 2018 Aug 7;9:1737. doi: 10.3389/fmicb.2018.01737 (PMC6090025; doi:10.3389/fmicb.2018.01737)
Supplement: Supplementary file 6 [file Table_6.pdf]

Table S6. Assignment of secondary structure components of *P. falciparum* PrLD amyloid core peptides in the amide I region of the FTIR spectra.

Sec24b		IF2c		PK4		
Band (cm^{-1})	Area (%)	Band (cm^{-1})	Area (%)	Band (cm^{-1})	Area (%)	Secondary structure
-	-	1610	16.5	1607	17.4	β-sheet
1630	47.6	1631	42.8	1633	44.8	β-sheet
1662	37.7	1662	40.7	1663	37.8	Random coil
1673	14.7	-	-	-	-	Loops/turns
