# Supplementary material for: Discovering Putative Prion-Like Proteins in Plasmodium falciparum: A Computational and Experimental Analysis
Source: Front Microbiol. 2018 Aug 7;9:1737. doi: 10.3389/fmicb.2018.01737 (PMC6090025; doi:10.3389/fmicb.2018.01737)
Supplement: Supplementary file 7 [file Data_Sheet_1.pdf]

Supplementary material references

Conchillo-Sole, O., de Groot, N.S., Aviles, F.X., Vendrell, J., Daura, X. and Ventura, S. (2007). AGGRESCAN: a server for the prediction and evaluation of "hot spots" of aggregation in polypeptides. BMC Bioinformatics. 8, 65.

Dosztanyi, Z., Csizmok, V., Tompa, P. and Simon, I. (2005). IUPred: web server for the prediction of intrinsically unstructured regions of proteins based on estimated energy content. Bioinformatics. 21, 3433-3434. doi:10.1093/bioinformatics/bti541

Fernandez-Escamilla, A. M., Rousseau, F., Schymkowitz, J. and Serrano, L. (2004). Prediction of sequence-dependent and mutational effects on the aggregation of peptides and proteins. Nature biotechnology. 22, 1302-1306. doi:10.1038/nbt1012.

Gasteiger, E., Gattiker, A., Hoogland, C., Ivani, I., Appel, R.D. and Bairoch, A. (2003). ExPASy: The proteomics server for in-depth protein knowledge and analysis. Nucleic acids research. 31, 3784-3788.

Tartaglia, G. G. and Vendruscolo, M. (2008). The Zyggregator method for predicting protein aggregation propensities. Chemical Society reviews. 37, 1395-1401. doi:10.1039/b706784b

Tsolis, A. C., Papandreou, N. C., Iconomidou, V. A. and Hamodrakas, S. J. (2013). A consensus method for the prediction of 'aggregation-prone' peptides in globular proteins. PLoS One. 8, e54175. doi:10.1371/journal.pone.0054175

Xue, B., Dunbrack, R. L., Williams, R. W., Dunker, A. K. and Uversky, V. N. (2010). PONDR-FIT: a meta-predictor of intrinsically disordered amino acids. Biochimica et biophysica acta. 1804, 996-1010. doi:10.1016/j.bbapap.2010.01.011

Yang, Z. R., Thomson, R., McNeil, P. and Esnouf, R. M. (2005). RONN: the bio-basis function neural network technique applied to the detection of natively disordered regions in proteins. Bioinformatics. 21, 3369-3376. doi:10.1093/bioinformatics/bti534
