# Supplementary material for: Discovering Putative Prion-Like Proteins in Plasmodium falciparum: A Computational and Experimental Analysis
Source: Front Microbiol. 2018 Aug 7;9:1737. doi: 10.3389/fmicb.2018.01737 (PMC6090025; doi:10.3389/fmicb.2018.01737)

Figure S1. Computational analysis of the Q/N-rich proteins devoid of PrLDs in *P. falciparum* proteome. Clusters of GO enriched terms for each ontology; biological process in blue, cellular component in green and molecular function in orange. The enrichment analysis was performed with Functional Annotation Tool of DAVID 6.7 database using high stringency clusters, p-value ≤ 0.05 for GO terms.

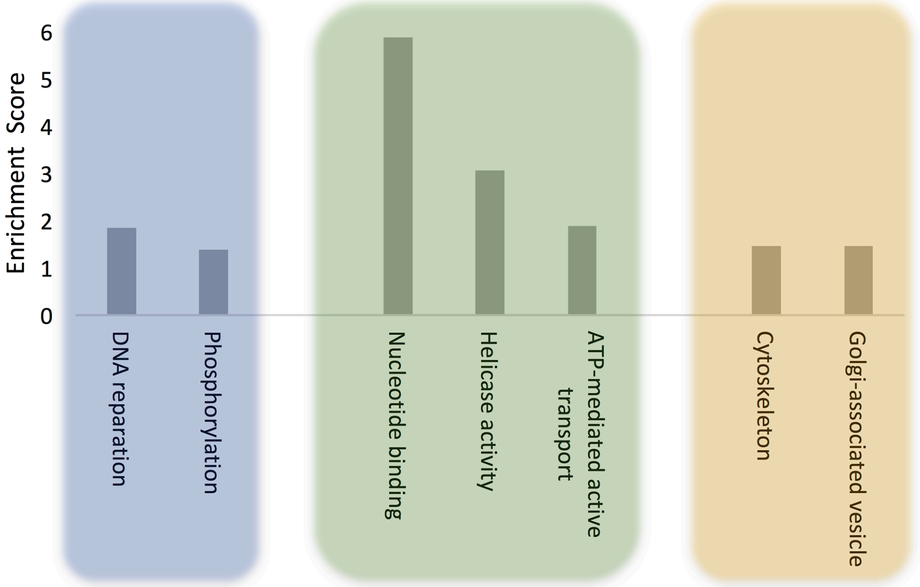

Figure S2. Size of *P. falciparum* Q/N-rich prion-like proteins. The size of 5353 and 503 proteins were analyzed and averaged for the complete proteome and the prion-like subset, respectively. The mean size of the proteins in the entire proteome is 764 ± 1193 residues. The mean size of the proteins in the prion-like subset is 1755 ± 59 residues. The P value for the unpaired t test < 0.0001.

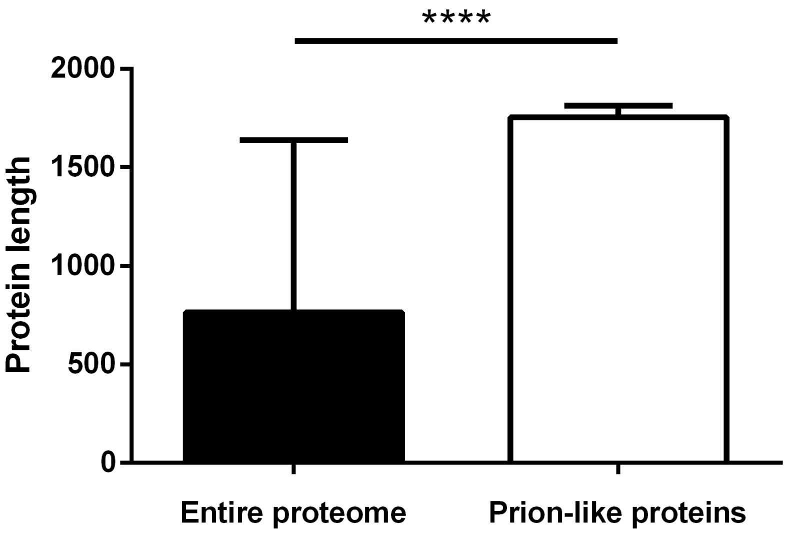

Figure S3. Developmental stages with highest prion-like protein expression. Highest expression stage for prion-like proteins in *P. falciparum*'s life cycle. Data corresponds to 10% highest scoring prion-like proteins (n=51).

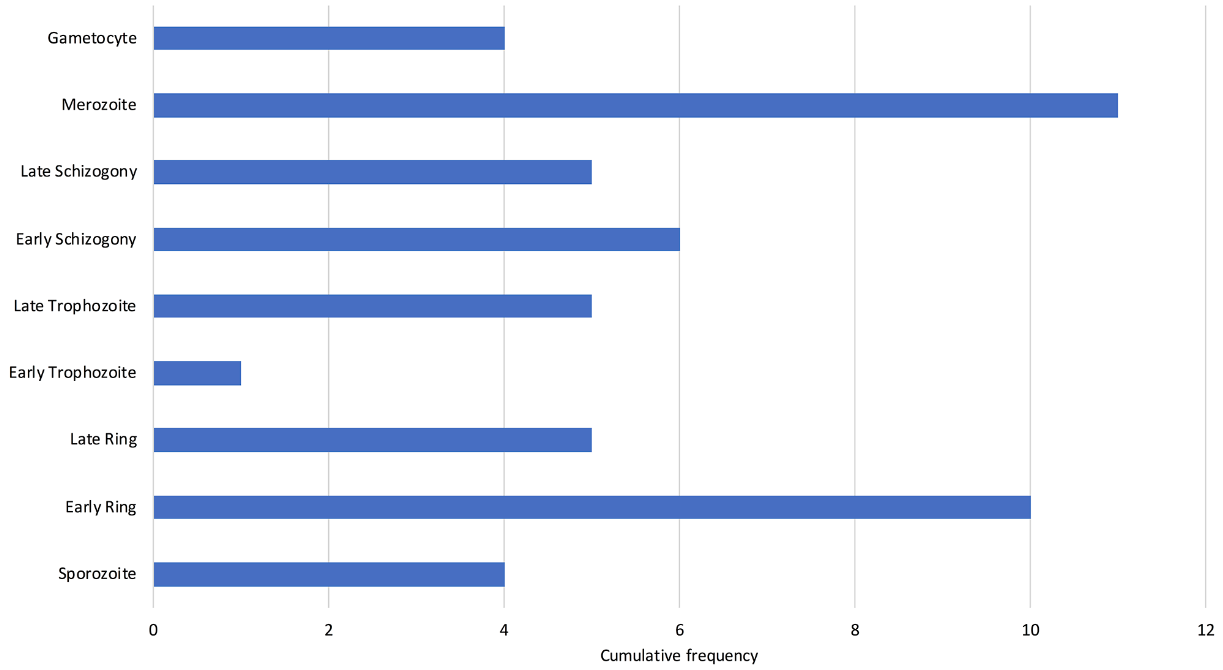

Supplement: Supplementary file 8 [file Image_1.pdf]
